# PRECOG: a tool for automated extraction and visualization of fitness components in microbial growth phenomics

**DOI:** 10.1186/s12859-016-1134-2

**Published:** 2016-06-23

**Authors:** Luciano Fernandez-Ricaud, Olga Kourtchenko, Martin Zackrisson, Jonas Warringer, Anders Blomberg

**Affiliations:** Department of Marine Sciences, Lundberg Laboratory, University of Gothenburg, Medicinaregatan 9c, 41390 Göteborg, Sweden; Department of Marine Sciences, University of Gothenburg, P.O. Box 461, SE 405 30 Göteborg, Sweden; Department of Cell and Molecular Biology, Lundberg Laboratory, University of Gothenburg, Medicinaregatan 9c, 41390 Göteborg, Sweden; Centre for Integrative Genetics (CIGENE), Department of Animal and Aquacultural Sciences, Norwegian University of Life Sciences, PO Box 5003, 1432 Ås, Norway

**Keywords:** Phenomics, Yeast, growth, Data pre-processing, Fitness components, Automation, Data presentation

## Abstract

**Background:**

Phenomics is a field in functional genomics that records variation in organismal phenotypes in the genetic, epigenetic or environmental context at a massive scale. For microbes, the key phenotype is the growth in population size because it contains information that is directly linked to fitness. Due to technical innovations and extensive automation our capacity to record complex and dynamic microbial growth data is rapidly outpacing our capacity to dissect and visualize this data and extract the fitness components it contains, hampering progress in all fields of microbiology.

**Results:**

To automate visualization, analysis and exploration of complex and highly resolved microbial growth data as well as standardized extraction of the fitness components it contains, we developed the software PRECOG (PREsentation and Characterization Of Growth-data). PRECOG allows the user to quality control, interact with and evaluate microbial growth data with ease, speed and accuracy, also in cases of non-standard growth dynamics.

Quality indices filter high- from low-quality growth experiments, reducing false positives. The pre-processing filters in PRECOG are computationally inexpensive and yet functionally comparable to more complex neural network procedures. We provide examples where data calibration, project design and feature extraction methodologies have a clear impact on the estimated growth traits, emphasising the need for proper standardization in data analysis.

**Conclusions:**

PRECOG is a tool that streamlines growth data pre-processing, phenotypic trait extraction, visualization, distribution and the creation of vast and informative phenomics databases.

**Electronic supplementary material:**

The online version of this article (doi:10.1186/s12859-016-1134-2) contains supplementary material, which is available to authorized users.

## Background

Thanks to recent technological innovations we can now detect and assess traits on virtually all phenotypic levels, from molecular to population level phenotypes, with unprecedented speed. These advancements have spurred the emergence of phenomics, the field in functional genomics that is dedicated the cataloguing of variation in phenotypes as a function of variation in genetic, epigenetic and environmental factors [1]. From a modest origin in documenting qualitative traits with moderate throughput, phenomics has evolved to precisely record quantitative traits with an astounding degree of parallelization. The massive accumulation of quantitative phenotypic data requires the continuous expansion of data storage, analysis, and visualization capabilities, something that has resulted in the development of an increasing number of specialized phenotype databases dedicated to the most important model organisms [2–7].

Microbes dominate the biosphere and microbial phenomics therefore has a key role in functional genomics [1]. A wide array of techniques have been introduced to address different aspects of the microbial phenome [8–21], but the growth of microbial populations and microbial growth phenomics takes centre stage because of its intimate link to microbial fitness and evolution. Microcultivation in liquid medium offers high resolution, but only moderate throughput, in the surveying of growth in microbial population size [1]. Recently, we and others have shown that microcultivation of microbial colonies on solid medium offers both high-resolution and high-throughput estimation of microbial population size growth [22–24]. Expectations are that this will lead to an explosion in microbial growth phenomics data and make activities in bioinformatics like storage, analysis and visualization of the accumulated information a critical issue. Various algorithms for analysing and extracting fitness components from microbial growth data have been developed for use in microbial growth phenomics [25].

Quantifying the growth in microbial population size at both high resolution and high throughput has technically two key components. First, an instrument that automatically and accurately records proliferation of a large number of microbial populations in parallel is needed. Several microcultivation instruments currently on the market achieve both a reasonable accuracy and a reasonable throughput [25–30]. The output from these instruments is files where the measured proxy for population size, e.g. optical density (OD), is listed for each experimental position as a function of time. Second, an analytical framework is needed to automatically handle, analyse and extract fitness components from each series of population size estimates and report the results in a convenient format. There is currently no generally accepted and widely used standard tool for the conversion of raw growth data into fitness components. On the contrary, most research groups develop their own ad hoc methods, leading to inaccurate, incomparable and incomplete data.

Algorithms to calculate growth rates, lag times and yield have been presented but are either not publically available [31, 32], or only available as Perl scripts [29] or Excel macros [25] upon request. GrowthRates, is a recently published, freely available software for analysing growth data [33] but does not contain visualization options for scrutiny of obtained curves in parallel to extracted traits. GATHODE, is a semi-automated open source software [34] that contains a graphical interface, but it is currently only available as source code and thus programming skills are essential for its installation. YODA, is mainly designed for chronological life span analysis and requires the establishment of a web-server [35]. Several of the above-mentioned tools lack the ability to correct for the non-linearity of the recorded optical density at higher cell densities, and none of them provide a quality measure of growth curves to speed-up and standardize the downstream analyzes of large data-sets.

To simplify analysis and standardize comparisons between different laboratories in the conversion of raw estimates of microbial population size into accurate fitness components, we developed the open source software PRECOG (PREsentation and Characterization Of Growth-data) that is ready to be downloaded in a desktop version, accessed via a web-site or utilized as a web-service (API). PRECOG converts the raw OD data into accurate population size estimates, provides quality indices, extracts fitness components, and presents both processed data series, i.e. the growth curves, and the extracted fitness components in a manner that allows easy user-interaction, exploration, and evaluation. PRECOG is designed to handle data generated by microcultivation in liquid cultures. However, PRECOG is essentially agnostic with regards to instrumental platform and requires no prior knowledge of the experimental design. This allows the user to initiate data-analysis immediately after the data acquisition has been completed. To enable proper comparison between research groups of experimentally generated traits it is essential to standardize procedures between laboratories at many levels, e.g. analytical procedures for data pre-processing and feature extraction [36]. PRECOG is a first step in our goal to create an extensive microbial phenomics framework that streamlines data acquisition and standardizes fitness component extraction and data visualization, storage and distribution.

## PRECOG implementation

**Fig. 1:**
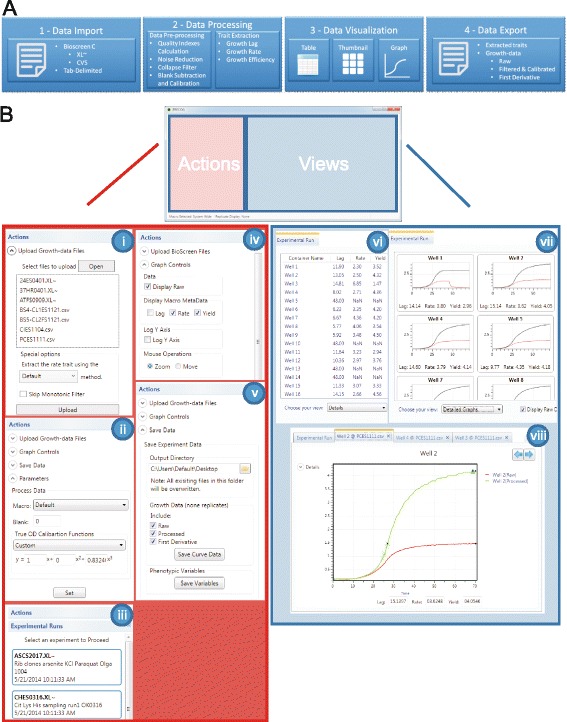
PRECOG’s overall design. **a** The functionality that PRECOG provides is organized as a pipeline that follows four basic steps: step 1 - data import, step 2 - data processing, step 3 - data visualization, and step 4 - data export. **b** Screenshots from the PRECOG desktop application. PRECOG’s user interface is divided into two zones: actions and views. The action zone controls the program’s functions: i) upload data files, ii) setting parameters, iii) experiment selection, iv) graph controls, and v) save data. The views zone presents the data in different displays: vi) table view, vii) thumbnail view and viii) detailed curve view

The PRECOG analysis pipeline consists of four steps (Fig. 1a): i) data import, ii) data processing, iii) data visualization, and iv) data export. PRECOG was initially developed to process time series of optical density data from the Bioscreen C instrument, but similar proxies of population size from other types of instruments can be used as input.

### System overview

PRECOG was implemented using C#, targeting the Microsoft.Net framework [37], and is accessible as three different platforms. A webserver hosts a website that operates as a gateway to PRECOG [38] and it can also can be reached via a link from our PROPHECY database [39]. The website hosts the three PRECOG platforms: a desktop application to be downloaded, installed and used locally, an online tool at the website and an Application Program Interface (API)(web service). A table comparing the functionalities of the different versions of PRECOG can be found in supplementary material (Additional file 1: Table S1). Documentation, a video-tutorial, access to publications linked to PRECOG, and contact details are also available at the website.

The desktop application can be installed on any Windows computer (Windows 7 or higher) from the website via the link “Desktop Application” on the main menu, or from the desktop install page [40]. Note that the program works without problems when ran on another operating system using a virtual machine with Windows installed (e.g. Oracle’s VirtualBox running on a Mac). The desktop platform offers the richest user experience by providing the most features, including an intuitive user interface, and rich graphics allowing the user to visualize, explore and evaluate the processed data. This publication is focused on the desktop application.

The online PRECOG platform, called PRECOG-lite, is a slimmed-down version of the desktop application that runs on a web-server. By connecting to the webpage, the user can upload a growth-data file and perform limited exploration of the extracted fitness components and the underlying population size growth curves. Finally, the user can download results as text files for further local analyses.

The API is a classic web service (responds to HTTP requests using the SOAP protocol) providing access to the code underlying raw data processing and fitness component extraction. This allows more advanced users to integrate the code employed into their own programs, regardless of operating system or programing language. The API service provides maximum flexibility but minimal visualization capabilities. The API service can be found at [41].

PRECOG will read an input file with several series of optical density data, pre-process each data series to remove technical noise and some technical bias, calibrate and convert the raw optical density data to actual population sizes, flag low-quality curves, extract fitness components and output both fitness components and the underlying data series (growth curves) for visual exploration and evaluation by the user.

All the core functions of PRECOG are stored in a virtual library referred to as the PRECOG engine. This core library is shared by all three versions of PRECOG.

### PRECOG desktop application

Using the Microsoft’s Windows Presentations Foundation (WPF) framework [42–44], we created a rich user interface for the PRECOG engine that functions as a stand-alone Windows client and facilitates the exploration and visualization of microbial population size estimates.

The user interface is divided into two zones: actions and views (Fig. 1b). The action zone organizes all the system operations under an “Actions Tab”. Here the user can find all the operations available in PRECOG as collapsible sections. When the user has imported data files in the “Upload experimental files” window, another tab on the actions zone, “Experimental Runs”, shows the uploaded growth-data files as individual experiments. Experiments can now be explored individually or in batch by selecting the relevant files. Exploration is performed in the views zone.

PRECOG has three basic user operations: Upload data files, Save data, and Parameters. The upload data section supports xl~ files and CSV exports generated by Bioscreen C instruments, and generic tab-delimited files. Unlike the Web and API PRECOG platforms, the desktop application allows processing and comparison of multiple data sets. The upload data section also allows the user to set special options, like the selection of the rate trait extraction method or the use of the monotonic filter. The parameters section lets the user customize the calibration function that converts the recorded optical densities into actual population size estimates. It also allows the user to specify the blank values that subtracts the background signal. At a minimum, the calibration function should be customized to each type of instrument and each species of microbes, and the blank values should be customized to each type of instrument and each medium. PRECOG allows the user to save and output data as tab delimited text files. Four types of data can be exported, as individual runs or in batches of more than one run. These are: raw unprocessed data, fully processed population size estimates, first derivatives (slopes) of the fully processed series of population size estimates and the fitness components extracted from each growth curve. Growth curves that were manually marked as excluded (see below) will not be included in the export files.

Once an imported data file is selected, data will be presented in the views zone. By default, it opens in the table view. This shows the fitness components extracted from each fully processed series of population size estimates. Data series are organized column-wise in the input file, with column headers being integer numbers, typically denoting position on the experimental plate, beginning from 1. Each data series is identified in the display by its column header (Fig. 1b). The table view supports full sorting and copy functionality, the latter allowing the user to copy from PRECOG and paste into other programs, e.g. MS Excel, for further analysis. This view shows the quality indices of each data series (data series and growth curve will here be used interchangeably) of fully processed population size estimates, supporting the user in decisions on to what degree the data series should be trusted. The user can then mark the data series for exclusion. It is also possible to merge multiple rows into a single graph. This feature is useful when wanting to visualize the effect of multiple samples at once. The user can change this view to a more compact display with many individual data series included in the same run as individual thumbnails, together with the fitness components extracted from each series (Fig. 1b).

A double click on a graph thumbnail creates a separate tab in the view zone with a detailed display of the sample’s growth curve (Fig. 1b). The detailed graph view allows the user to interact with the data in detail; the user can perform zoom and pan operations, use logarithmic or non-logarithmic *y*-axis, and simultaneously visualize both the raw optical densities and the fully processed population size estimates. The system also shows markers in the region of each data series where the fitness components were extracted as metadata (Additional file 2: Figure S1). This allows the user to visually detect and avoid the introduction of analytical bias that could confound fitness component extraction. The detailed graph view has another useful function; it can show the first derivative of the curve. The first derivative maximum should overlap with the growth rate metadata markers (Additional file 2: Figure S2). If not, this is a clear indication of analytical errors in the fitness component extraction. The detailed graph view, its underlying data and the extracted fitness components can be copy-pasted into other applications.

### PRECOG-lite – online version

The online PRECOG-lite platform is a slimmed-down version of PRECOG that allows the user to upload a single experimental file. The raw optical densities will be pre-processed and calibrated as in the desktop application and the fully processed population size estimates will be presented as growth curves. These can be saved in a tab-delimited text file. During the upload and process steps, the user can set several parameters like the ones determining the calibration function, the blank value, the growth rate trait extraction method and if they want to skip the monotonic filter (Additional file 2: Figure S3). Once the data has been processed, it can be accessed in the same three formats as in the desktop application: table view, thumbnail view and detailed graph view (Additional file 2: Figure S3). In the online version, potential low-quality curves are flagged for visual inspection by one or more of the four quality-indices. However, the user cannot in PRECOG-lite mark the curves for exclusion as can be done in the desktop version.

### PRECOG web service – API

The API version of PRECOG offers access to some of the basic functions of the core library (see [45] for an overview), allowing other developers to use them in their own programs. The current API implementation is basic and is intended to evolve organically following user input.

### Pre-processing filters reducing noise and bias

**Fig. 2:**
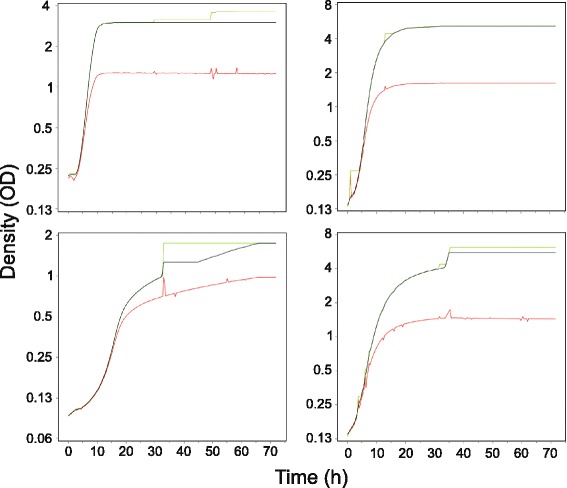
Effects of data pre-processing. Effects of different types of noise in the raw data (*red line*) on the fully (*black line*) or partially (*green line*) processed data, the latter without the mean filter that removes spikes. If spikes are not removed, as in the case of wide spikes consisting of more than one data point, the processed data will be distorted (as seen in the lower two graphs). Figures are screenshots from PRECOG.

To remove or reduce random noise and technical bias, PRECOG filters the raw optical densities in a three-step procedure. This is done before the background subtraction and the calibration that transform raw optical densities into actual population size estimates. First, a sliding window one-dimensional median filter considers each three consecutive data values in the data series, replacing the middle value with the median of all three. Edge cropping is avoided by padding (filling the gap by cloning the endpoints). Median filters are standard to minimize the impact from high amplitude noise at single time points, “spikes”, while preserving the overall data characteristics [46–49]. Optical density spikes originate from lamp failure, heterogeneity in the cell population, electronic interference, dust particles or sudden change in light influx from the environment. In the evaluated test data series, spikes affected about 4 % of 90,000 growth curves. Whereas the median filter effectively removed spikes at single time points (Fig. 2; upper two graphs), it failed to remove the rare “wide spikes” that extended over multiple consecutive time-points (Fig. 2; lower two graphs).

Low amplitude but high frequency noise is challenging to remove. Standard signal analysis instead seeks to minimize the influence of such noise by smoothing series of signals, accepting that also data that is close to the truth are subject to minor adjustments [47–50]. After the median filtering, PRECOG therefore employs a one-dimensional mean filter in a sliding window that considers three consecutive data values. The middle value in each window is replaced by the mean over the three values in the series. The smoothing is among the lightest employed in signal analysis, accepting that some noise may penetrate in order not to distort the true data trends.

Neither the median nor the mean filter is capable of accounting for the strongly confounding noise and bias associated with collapsing growth curves. Curve collapses follow from the sudden emergence of dramatic heterogeneity in microbial populations, typically due to bubble formation, cell-cell cohesion leading to aggregates, or well - wall adhesion leading to deprivation of cells in the central well area. Curve collapses are associated with drastic drops in cell density, often followed by equally drastic increases when bubbles and cell aggregates drift stochastically in and out of light beams. PRECOG, as a third filtering step, sweeps each data series and enforces data monotonicity by replacing any values lower than its predecessor with the predecessor itself (Fig. 2). Given that the enforcement of monotonicity has been preceded by both median and mean filtering, its effects are restricted to removing or reducing abnormalities in collapsing curves. A relevant note of caution is that there might be naturally occurring biological phenomena that result in a negative net growth (curve collapses), e.g. bacterial cultures being treated with cell lysing agents like certain antibiotics, or being exposed to changing osmotic pressure or starvation, with associated cell lysis and/or autophagocytosis. To make possible studies of these phenomena using PRECOG, there is an option where the monotonicity filter can be excluded.

### Background/blank subtraction and data calibration

The last pre-processing steps before the fitness components are extracted are the blank subtraction and the calibration adjustment. The blank value, provided by the user, is subtracted from every data point in the data series. This effectively removes the background signal on the assumption that the background is constant across all experimental positions. Recorded optical densities do not increase linearly with cell densities because of an accelerating cell shielding effect against light at higher densities. This is typically detectable at OD > 0.3 [25]. Sample dilution is not logistically feasible in high-throughput growth phenomics; thus, recorded optical densities have to be analytically transformed into actual population size estimates. The transformation uses an empirically established calibration function that should be separately established for each species and instrument type. For the Bioscreen C instrument, a calibration function has been calculated and applied to correct for this effect. The function is valid for most lineages and physiological states of the model yeast *Saccharomyces cerevisiae* [25] and is offered as default.

### The default procedure for fitness component extraction

The traditional view of the microbial population size growth curve postulates an initial lag phase of no net increase in cell number, an exponential phase where net increase in cell number is positive and constant and a stationary phase of no net increase. These phases are separated by acceleration and deceleration phases that typically are disregarded in the analysis. The inevitable final phase of net death, which rarely is initiated within the time-frame of a typical experiment, also tends to be overlooked. In this traditional view, the three fundamental fitness components is the length of the lag phase, the rate of growth in the exponential growth phase and the total gain in population size up until growth ends in the stationary phase. The latter reflects the efficiency with which the limiting resource has been converted into population size growth. PRECOG extracts these three fitness components, using the algorithms earlier presented [25], in the following way:

The growth lag is extracted from population size estimates, presented on the log-scale as the intercept between the initial population size and the line extrapolated from maximum growth. PRECOG calculates the mean of the first five population-size estimates, providing a robust measure of the initial OD. PRECOG then calculates the time for all the intercepts between the initial mean values and every slope. Slopes are obtained in a moving window of eight time-points, departing from the beginning of the curve and proceeding to the end. The fitness component lag time is estimated as the mean of the two largest intercepts and is expressed in hours.

The growth rate, expressed as population size doubling time, is extracted from the highest of many short slopes in the exponential growth phase and converted into population doubling time. The first three hours of the curve is excluded to avoid obtaining slopes corresponding to cell size increases when cells exit from the starved G0 state and recapture a size that is permissive for cell division. Then it takes the logarithm (base 10) of the remaining data points and calculates the slopes in a moving window of three time-points. Slopes are ranked based on their values and the two highest slopes are discarded in order to minimize the effect of any remaining artefact outliers. From the remaining slopes, the mean of the five highest is formed $\left({\overline{x}}_{highest\  slopes}\right)$ and converted into doubling time (in hours).

The growth efficiency is extracted as the total increase in population size, from the smallest to the largest population sizes, given that growth has actually ceased when population size is maximal. The mean of the two smallest measurements $\left({\overline{x}}_{base}\right)$ and the mean of the six highest measurements $\left({\overline{x}}_{top}\right)$ defines the interval such that the efficiency is estimated as ${\overline{x}}_{top}\kern0.5em -\kern0.5em {\overline{x}}_{base}$. No estimate of efficiency is given if the standard deviation of the six highest OD measurements divided by $\left({\overline{x}}_{top}\right)$ is greater than 0.02, corresponding to a rough distinction between curves that have and have not reached a stationary phase.

PRECOG also provides an alternative algorithm for growth rate extraction that is less prone to overestimate the growth rate when growth curves are noisy and more robust when data are sparse. The algorithm performs linear regressions in a window of five time points that slides along the curve, extracting slopes from the regression models and converting the highest slope into doubling times.

### Quality indices

Automatic evaluation of the quality of the data series of population size estimates is challenging because both high-quality data series, that should be retained, and low-quality data series, that should be rejected, vary greatly in their growth dynamics and types of technical errors (Additional file 2: Figure S4, Figure S5). To distinguish high from low quality growth curves given this variability, PRECOG establishes four quality indices (QI), based on the raw data series, that together estimates curve quality. The indices are QI1, “overall noisiness” in the form of the average fit of regression lines to the data along the curve, QI2, “local noisiness” based on local regions with poor fit of regression lines to the data along the curve, QI3, “number of spikes” that identifies short, dramatic increases in data values and QI4, “curve collapses” that identifies curves with sudden and sustained drops in data values. Conceptually, these phenomena are all incompatible with accepted models of true growth curve dynamics. High values in one or more of the quality indices therefore suggest technical problems and identify data series that should be visually inspected by the user for possible exclusion.

For the QI1, “overall noisiness”, we exclusively consider the noise in the growth phase of the curve, by calculating linear regression over a five-point sliding window with slope *k* > 0.07. QI1 is defined as 1 - the average coefficients of determination, *r*^2^, of the data in all such windows to their respective linear regression, and provides a measure of the overall noisiness in a data series.

The QI2, “local noisiness” uses the same principle as QI1 but only considers high local variation of the growth data. QI2 is defined as 1 - the average of the three worst coefficient of determination, *r*^2^, of the linear regressions. Thus, it indicates the existence of very noisy regions.

The QI3, “number of spikes”, estimates the number of spikes in the growth curve. The QI3 index is initially calculated in the same way as the QI2. However, here PRECOG counts the number of *r*^2^ values that are less than 0.5. From practical experience we have seen that this index shows how many sharp bends the curve has, thus being an indication of the number of spikes.

The QI4, “curve collapses”, measures the magnitude of the corrections done to the raw growth curve. The QI4 index is calculated by comparing data series before and after filtering with the monotonic filter. We obtain QI4 as the sum of the absolute differences between the data series, over all time-points. Large correction values reflect curves that have collapsed in late exponential or stationary phase. These are rarely captured by other quality indices.

## Methods

### Establishing the calibration function

To make the species-specific calibration functions to correct higher OD values, cells were grown overnight to stationary phase in synthetic defined media; YNB with 2 % (w/v) glucose for the various yeast species and LB medium for *Escherichia coli*. The stationary phase cultures were x1.5 serially diluted in the corresponding growth media to finally reach 15 dilution-steps, and the whole series of dilutions measured in the Bioscreen instrument. The blank-corrected diluted OD values and the blank-corrected undiluted OD values are for practical purposes indistinguishable up to OD ≈ 0.3, and above this value the true OD values were calculated using the corresponding dilution factor and the diluted samples (OD < 0.3). A blank value was estimated on only media without cells, and this value was subtracted from all other measured OD values. Using the previously established formula for calibration, y = x + cx^3^ [51], which assumes a near 1:1 linearity for low OD, the following values for c were found: *Candida albicans*: c = 0.5790256635480614, *Pichia pastoris*: c = 0.5653284345804932, *Schizosaccharomyces pombe*: c = 0.64672463774234579, and *E. coli*: c = 0.75389848795692815. For *S. cerevisiae* we obtained c = 0.82673123484708266 which is very close to the earlier established and since long used c value for this species, 0.8324057 [19, 51]; for consistency reasons we will stay with the old c value.

## Results

### Flagging low-quality data series for manual inspection and exclusion

To evaluate the performance of PRECOG, we created two benchmarking sets of 100 high- and 100 low-quality growth curves. Curves in the high-quality set are technically of good standard and capture a wide range of true biological properties, including individual and combined effects on each of the classical fitness components growth lag, rate and efficiency, as well as multimodal growth. Multimodal growth corresponds to multiple phases of elevated growth (Additional file 2: Figure S4 and data S1). Growth curves in the low-quality set represent a variety of amplitudes and frequencies of technical noise (Additional file 2: Figure S5 and data S2); roughly half of the curves in this benchmarking set are completely dominated by technical error and should be rejected. We believe that these two benchmarking sets could be generally valuable in microbial phenomics for performance tests of growth algorithms and software. The two benchmarking sets can be downloaded from [52].

Growth curves are quality filtered by PRECOG in a semi-automated procedure. The first step is the automatic calculation of quality indices based on four curve features. The second step is manual scrutiny of indicated problematic curves for possible exclusion. The four quality indices are QI1; “overall noisiness”, QI2; “local noisiness”, QI3; “number of spikes” and QI4; “curve collapses”. Thus, the four indices capture different types of technical problems encountered, and one should not expect all indices to flag for low quality of a particular problematic curve. Low-quality data series evading the four quality indices may result in extractions of incorrect fitness components. On the other hand, false flagging of high-quality data series results in an excessive workload on the operator, in terms of manual inspection.

**Fig. 3:**
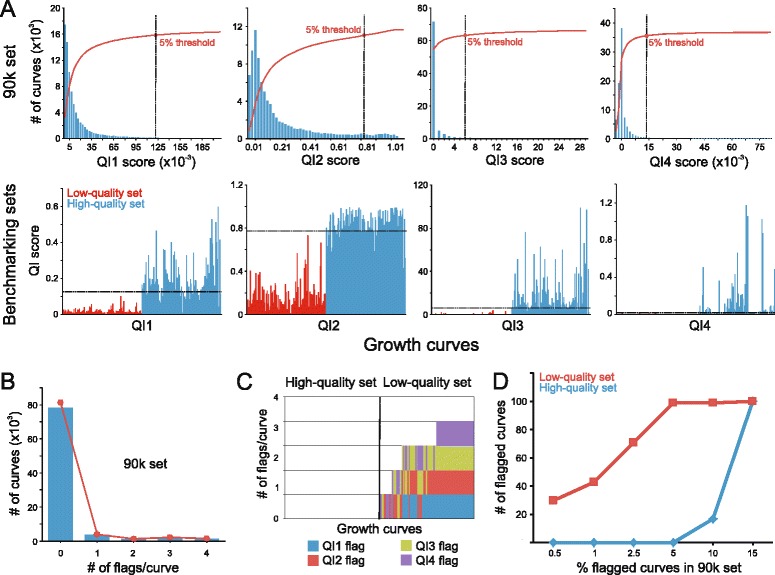
Filtering data using PRECOG’s quality indices. **a** Data is filtered using four quality indices, QI1 - “overall noisiness”, QI2 - “local noisiness”, QI3 - “number of spikes”, and QI4 - “curve collapses”. Upper panels: performance of each quality filter on the “aggregated 90 k set”, including almost 90,000 growth curves. *x*-axis shows QI score, *y*-axis shows number of growth curves flagged at each QI score setting. Blue bars = non-cumulative flagging, red line = cumulative flagging, dashed black line = selected QI score setting that flags a cumulative 5 % of curves. Lower panels: performance of the QI filter (QI score, *y*-axis) on the two selected benchmarking sets of 100 high- and 100 low-quality curves (*x*-axis). Dashed horizontal lines: performance at the 5 % rejection threshold selected based on the “aggregated 90 k set” growth curves. **b** Summary performance of all quality indices. Number of curves that obtain 0, 1, 2, 3 and 4 flags in the “aggregated 90 k set” at the selected threshold, where each quality index flags the worst 5 % of growth curves, i.e. 90 % of all curves were not scored by any of the quality indices while 2 % were scored by all four. **c** Summary performance of all quality indices. Number of QI flags in the high- and low-quality benchmarking sets. Colours indicate quality index responsible for the flagging, with blue = QI1, red = QI2, green = QI3 and purple = QI4. **d** Number of false positives and negatives in the two benchmarking sets, as a function of using various thresholds from the “aggregated 90 k set”

To find the optimal breakpoint for what to flag and what not to flag, we evaluated six thresholds for each quality index: flagging the worst 0.5, 1, 2.5, 5, 10 and 15 % of data series on almost 90,000 growth curves, the aggregated 90 k set, generated over many years and by several different experimentalists in our lab (Fig. 3). Each threshold was then tested on the two benchmarking sets to evaluate false positives and false negatives. We found the numbers of false positives, in the high-quality set, and false negatives, in the low-quality set, to be lowest at the 5 % flagging level for each QI in the aggregated 90 k set (Fig. 3d). At this threshold the low-quality curves were easily distinguished by the quality indices; only one curve escaped all four quality-indices. None of the high-quality curves were flagged at the 5 % threshold. It was also clear that different quality indices captured different curve problems (Fig. 3c); only 39 % of the low quality curves were flagged by all four quality indices while roughly 11 % were captured by only one quality index. The flagged curves can be visually inspected in PRECOG in a semi-automated manner, allowing fast operator decisions on which flagged curves to accept and reject. Non-flagged curves are automatically accepted and included in the downstream analysis.

### Benchmarking PRECOG’s pre-processing data filters for minimizing noise

**Fig. 4:**
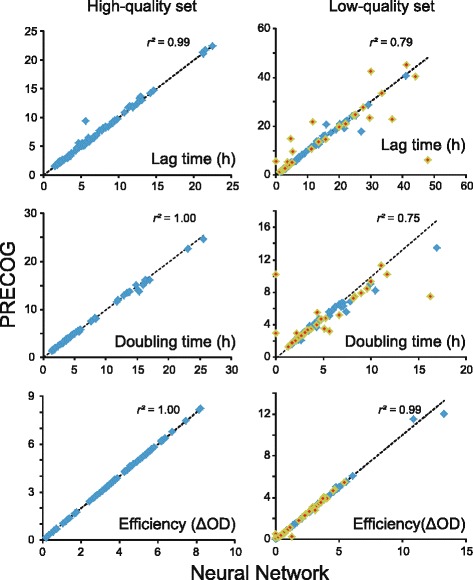
Benchmarking of PRECOG’s default data cleaning algorithm. We compared PRECOG’s pre-processing filters against a computationally demanding neural network pre-processing procedure. After the pre-processing, the fitness components growth lag, growth rate and growth efficiency were extracted by PRECOG’s standard procedure from the high- (left panel) and low-quality (right panel) benchmarking sets, and compared. Low-quality curves are in the right panel marked (green with a red mark) if flagged by all four quality-indices (thus, indicating curves of really low quality)

To remove or reduce noise and bias, PRECOG filters the raw optical densities in a three-step procedure. PRECOG’s data filtering is computationally inexpensive and therefore possible to employ on a very large scale, and addresses the vast majority of technical errors manifesting in data series. We tested PREGOG’s data filtering by comparing its performance to a multi-layered neural networks noise reduction filter. Neural networks have been used for many years as a reliable tool to solve noise reduction problems [53, 54]. A multi-layered neural network, iterated 500x to exclude outliers (remove noise), did not perform better than the three-step PRECOG data filtering process (Fig. 4). Fitness components, extracted following the data processing by the two methods, were nearly identical for high-quality curves (*r*^*2*^, lag = 0.9938, rate = 0.9976, efficiency = 0.9997) and correlated well also for low-quality curves (*r*^*2*^, lag =  0.7868, rate =  0.7465, efficiency = 0.991); if the really bad curves flagged for all four QI:s were excluded the correlations were even better (*r*^*2*^, lag = 0.9716, rate = 0.9762, efficiency = 0.9933). Visual inspection of low-quality data series where noticeably different outcomes were observed, failed to establish a pattern of consistent superior performance of any one filtering method. The computational inexpensiveness of PRECOG’s three-step data pre-processing therefore speaks emphatically in its favor.

### The importance of calibration to convert optical densities into population size estimates

**Fig. 5:**
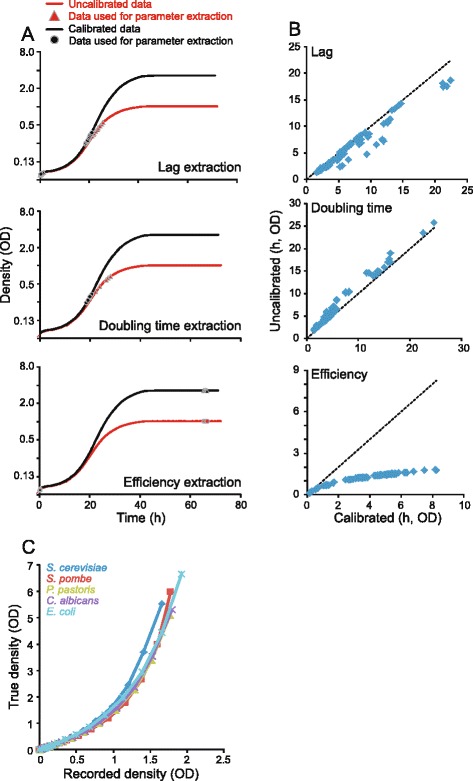
Fitness components extraction from calibrated and non-calibrated growth curves. Fitness components were extracted from the high-quality benchmarking set of growth curves. **a** Example curves for each of the fitness components extracted (growth lag, rate and efficiency). For each estimated fitness component, markers (red triangles = non-calibrated data, black circles = calibrated data) indicate the data underlying that estimate. **b** Correlation between calibrated and non-calibrated data. Dotted line indicates the 1:1 relation. **c** Calibration function for different organisms. Recorded optical density (*x*-axis) and actual population size (density), as reflected in the OD recorded for a diluted cell suspension and multiplication with the dilution factor (*y*-axis), is shown

The optical density of cultures is easy to measure and can be implemented in automated procedures, which is why it has become a standard way of estimating changes in high-throughput microbial population size studies. Calibration of recorded optical densities into population size estimates drastically adjusts the shape of recorded growth curves, suggesting a potentially profound distortion of fitness components extracted by softwares failing to employ this procedure (Fig. 5). To evaluate to what degree fitness components are confounded by failure to calibrate, we compared fitness components extracted from the high-quality benchmarking set, before and after calibration (Fig. 5b). Distortions in the form of a systematic underestimation of lag phases, corresponded to 15 % shorter lags than reality, and systematic overestimation of population doubling times, corresponded to 25 % slower growth than reality, were substantial. In the case of growth efficiency, the systematic underestimations from non-calibrated data were outright catastrophic, with a mean of 61 % lower estimates than reality (Fig. 5b). Thus, calibration of recorded optical densities is absolutely essential and should never be overlooked.

PRECOG allows the user to choose between calibration functions stored centrally or to enter functions established empirically by the individual laboratory. It is clear from our experimental data that different organisms require different calibration functions (Fig. 5c). For example, the relation between observed optical density and actual population size is very different in the fission yeast *S. pombe* than in the budding yeast *S. cerevisiae*. In this context it should be mentioned that OD is influenced by other factors besides cell number, like the volume and biomass of each cell, absorption from internal molecules, the shape of individual cells and cell aggregation. This means that OD measurements are most effective in comparing microbial cultures for which other properties that affect light transmission are identical. Thus, OD should always be seen as a proxy for the microbial population size, and interesting phenomena should ideally be confirmed by alternative cell-counting methods, like microscopy or FACS, for which the biases are different. The impact from the calibration function will especially be great for the extraction of growth efficiency, as calibration functions deviate more from linearity at higher optical densities. PRECOG enables users to enter calibration functions established by comparing optical densities of diluted and non-diluted microbial samples over a range of densities [25] (see Methods section for a more detailed description of experimental and analytical procedures for establishing valid calibration functions).

### Fitness component extraction and frequency of sampling

PRECOG extracts the three canonical fitness components associated with net growth of microbial populations; length of the initial lag phase, in which no net growth occurs, maximal rate of growth (specifically: minimum population doubling time) in the phase when net growth is positive, and the total gain in population size, given that a stationary phase of no further net growth has been reached at the maximum population size. Stationary phase is almost exclusively entered because one resource, typically energy or nitrogen, has been depleted, and the population size yield therefore represents the efficiency with which this resource has been converted into population growth. PRECOG also tracks the data points used to extract each fitness components as associated metadata, allowing stringent user evaluation of the reliability of each extracted fitness component (Additional file 2: Figures S1, S2).

**Fig. 6:**
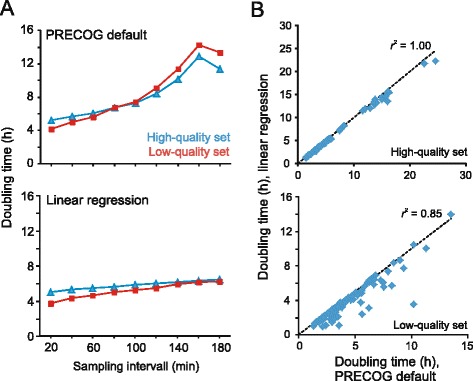
Comparing two algorithms for extracting doubling time. **a** Effect of sampling frequency on doubling time. Sampling frequency denotes the fixed time (interval) between consecutive measurements. At the start of the experiment the user sets the sampling frequency: PRECOG’s default algorithm (upper panel), the algorithm based on linear regression (lower panel). Averages from the high- and low-quality sets are indicated. **b** Doubling times extracted from data with 20 minute sampling intervals (our default value) for the high- (upper panel) and low-quality (lower panel) benchmarking sets are shown for the two algorithms

The primary experimental settings that operators need to decide on prior to experiment start are frequency of data measurements and total number of data measurements. Together, these parameters define the length of an experiment. We evaluated the influence of varying the data measurement frequency on extracted fitness components in a standard 72h experiment, using the two benchmarking sets. While growth efficiency and lag were largely unaffected by realistic frequency changes (Additional file 2: Figure S7), minimum population doubling times were systematically and continuously overestimated when data measurements were less frequent (Fig. 6a, upper panel). Minimum population doubling times typically occur early in the net growth phase. As data measurement frequency decreases, minimum population doubling times are increasingly based on data points from later in the net growth phase, where slopes are less steep and the exponential growth assumption increasingly incorrect (Additional file 2: Figure S6).

We also tested an alternative method for rate extraction, based on a single linear regression over five consecutive time-points, expecting it to have a lower degree of dependency for alterations in frequency of sampling. Indeed, we found that the tendency towards overestimating true minimum population doubling times at lower measurement frequencies was somewhat less severe when linear regressions over five consecutive time-points were considered (Fig 6b). Comparing the two growth rate extraction methods on high frequency sampled data, we found near perfect correlation (*r*^2^ = 0.9966) over the whole range of growth rates in the high-quality data set (Fig. 6b), but a slight, systematic tendency for extraction of shorter minimum population doubling times using linear regression. This tendency was substantially stronger in the low-quality data set, where the correlation was also less impressive (Fig. 6b; lower graph). The discrepancy between the two methods primarily originated from the default method, based on the mean of many high slopes, discarding the two highest slopes; when including the two highest slopes in the growth rate extraction, the correlation between methods was excellent (*r*^2^ = 0.9705) and the systematically faster growth extracted using linear regression disappeared (data not shown). We conclude that for high-frequency sampled growth data, maximal population doubling time estimates are mostly independent of which of the two methods are used. In PRECOG the user can select the preferred growth rate extraction method.

## Discussion

Estimation of phenotypes is central in experimental life sciences. Advancing the speed and accuracy with which phenotype estimation can be achieved is therefore critical to scientific progress. Although phenomics still lags behind genomics [55], the throughput of phenotypic data acquisition has now, for a wide set of model organisms, advanced to a degree that data analysis, standardization, visualization and validation are becoming critical bottlenecks. The computational challenge is amplified by that many phenomics instruments, each with their own noise levels, biases and limitations that need specific attention, are in use without unified industry standards [36]. Concerns over the reproducibility, comparability and accessibility of the vast resources of phenotypic data are also being voiced by an increasing fraction of the life science community [56–59].

Variation in experimental designs in terms of genetic background, environmental factors, media composition and instrumental set-up is essential, both for exploring the full width of biological phenomena and for establishing the general validity of conclusions across wider swaths of the experimental space. This variation is beneficial and leads to that more of the phenotypic space is examined. Nevertheless, standardization in the downstream analysis is required in order to allow strict comparisons across platforms, verification of the reproducibility and general validity of conclusions, and simplifying data mining for third parties. We hope that PRECOG can play such a standardising role.

Currently, phenotypic data is mostly presented using free text combined with controlled vocabularies, like the Yeast Phenotype Ontology [60]. However, the qualifiers used, e.g. arrested, delayed, decreased, increased, and normal, imply the direction of change relative some type of reference but lacks quantitative definitions in terms of effect-sizes, the confidence with which conclusions have been established, and the conditions under which they were found to be true [4]. This certainly leaves much room for improvement [61], including the use of proper references, quality assessment of data, calibration, standardized software data processing and fitness component extraction, and correctly accounting for noise and bias.

PRECOG is first and foremost an effort in the direction of standardized data processing and fitness component extraction. Additionally, PRECOG aims to simplify data analysis, visualization and evaluation for users that lack the experience, time, and computational expertise to develop the tools required to achieve these tasks. Most commercial software dedicated to analysing growth curves have built-in capacities to extract various variables, such as the maximum signal intensity, the maximum slope, or the integral under the curve. Unfortunately, these built in software features typically extracts variables from raw growth curve data, i.e. without any calibration to obtain true estimates of population size and without smoothing of population sizes to minimize noise and bias. As shown in Fig. 5, this will result in grossly misleading results. Moreover, the specifics of the procedures for variable extractions are rarely published for proprietary reasons and are not comparable across either programs or versions of programs, leading to severe issues with reproducibility between various laboratories and over time. PRECOG with its implemented calibration functions and transparent feature extraction procedures thus facilities automated processing of microbial growth phenomics data in a manner that provides high accuracy, minimizes risks for false positive and negatives, allows the user to visualize and evaluate individual data series such that extracted fitness components can be directly connected to the underlying growth curves, and provides easy and standardized exporting functions. Overall, we found that the established analysis pipeline handles high-quality curves well, with no operator intervention needed, whereas low-quality growth curves pose challenges that cannot easily be overcome in an automated fashion. We partly solved this by flagging low-quality data series for operator inspection and potential rejection, using a series of quality indices. We delegate the decision to the operator as to what is the best overall strategy, with regards to rejecting or accepting questionable data series. Ideally, experiments should be designed with sufficient replication such that rejection of even a rather high fraction of growth curves as being of too low quality would not challenge the statistical integrity of the analysis. In reality, few experimental designs are replicated at a level where rejecting of substantial amounts of data series can be done without painful costs. This calls for further advancements in throughput, and perhaps a refocusing from number of distinct samples to number and randomization of replicates of these samples. Downstream analysis of obtained growth data will naturally involve sound statistics. Certain replicates might in these statistical analyses be scored as outliers; if these correspond to curves showing quality warnings that would be an excellent base for exclusion.

As with any method, there are room for future improvements of the PRECOG platform. We currently make poor use of the richness of the data acquired, restricting fitness components extraction to the lag, rate and efficiency of growth. These parameters only reflect a fraction of the growth information in the data accumulated. Focus on these fitness components are partially motivated by the established standard model of growth, assuming distinct lag and stationary phases that are separated by a long exponential phase. This standard model is at best a very crude approximation of a complex and very diverse reality, where growth often rapidly becomes limited by external factors and therefore switches from exponential to linear growth, or where serial use of nutrients or handling of toxins promotes diauxic rather than unimodal growth [62]. Fitness, in terms of a genotypes’ frequency increase relative other genotypes, are naturally affected by its performance in each of these phases. Fitness component extraction would therefore certainly benefit from a conceptual re-think. Full exploitation of the first derivate of growth is a natural first step in this direction. Expanding fitness component extraction requires careful attention to the particular biases that affect the different phases of growth, with systematic differences between measurement positions on an experimental plate and the relative impact of different normalization procedures applied to account for these constituting key question marks.

## Conclusion

To summarize, we here launch the tool PRECOG that will promote simplicity, transparency and standardization in microbial growth phenomics, and provide a portal with the standalone software and online tools so that other microbiology labs can easily upload, assess and visualize their growth data. Data may then be further distributed to standard repositories, like the SGD [4] and PROPHECY databases [63, 64].

## Availability and requirements

Project name: PRECOG

Project home page: http://precog.lundberg.gu.se/

Operating system(s): Desktop Application: Windows; Online version: Platform Independent; API(Web service): Platform Independent

Programming language: Microsoft C#

Other requirements: Dot Net framework 4.5 or higher

License: EULA

Any restrictions to use by non-academics: Software is free for non-commercial users.

The high-quality set is included as Data S1.

The low-quality set is included as Data S2.

## Additional files

Additional file 1: Table S1. Feature comparison of the different PRECOG platforms. (DOCX 17 kb) Additional file 2: Figure S1. Meta data to evaluate the extracted fitness components. Markers indicate data used for estimation of growth lag (purple circles), rate/doubling time (black cross), and efficiency (green triangles). Figures are screen shots from PRECOG. **Figure S2**. Displaying the first derivative of growth. X’s mark the samples where the doubling time was extracted, which coincides with the first derivative peak. Allows the user to identify curves where there are difficulties in extracting traits, like curves exhibiting multimodality (B and C). Figures are screenshots from PRECOG. **Figure S3**. PRECOG-lite website screenshots. (A) upload, B) table view, C) thumbnail view, D) a detail view of the growth-data, E) the Save As allows the user to save the data in its different forms (growth-data, first derivate, and extracted traits). **Figure S4**. High-quality benchmarking set of growth curves. The 100 growth curves of high quality displaying various growth feature, i.e. difference in growth lag, rate and efficiency as well as combinations of these. The set also include curves that are clearly multimodal. Red = raw data, Black = fully processed data. All curves are displayed on the log scale (y-axis). **Figure S5**. Low-quality benchmarking set of growth curves. The 100 growth curves of low quality, representing various technical challenges, e.g. curves with high levels of noise, frequent spikes and collapsing curves. Red = raw data, Black = fully processed data. All curves are displayed on the log scale (y-axis). **Figure S6**. First derivative of growth curves with various sampling frequencies. First derivative is displayed indicating times for measurements (red circles), and data used for estimation of growth rate (crosses). **Figure S7**. The effect of sampling frequency on growth lag (upper panel) and efficiency (lower panel). Sampling frequency denotes the fixed time (interval) between consecutive OD measurements. Averages from the high- and low-quality sets are indicated. (PDF 452 kb)
