# Supplementary material for: PRECOG: a tool for automated extraction and visualization of fitness components in microbial growth phenomics
Source: BMC Bioinformatics. 2016 Jun 23;17:249. doi: 10.1186/s12859-016-1134-2 (PMC4917999; doi:10.1186/s12859-016-1134-2)
Supplement: Additional file 1: Table S1. — Feature comparison of the different PRECOG platforms. (DOCX 17 kb) [file 12859_2016_1134_MOESM1_ESM.docx]

**Table SI Feature comparison of the different PRECOG platforms**

| Features | **Desktop** | **Web** | **API** |
| --- | --- | --- | --- |
| **Requirements** | | | |
| Internet access | No | Yes | Yes |
| Platform | Windows OS | OS independent | OS independent |
| Programing experience | No | No | Yes |
| **Actions** | | | |
| Upload Bioscreen and Generic formats | Yes | Yes | Yes |
| Process uploaded growth data | Yes | Yes | Yes |
| Choose rate extraction method | Yes | Yes | No |
| Skip Monotonic Filter | Yes | Yes | No |
| Select a pre-set calibration function | Yes | Yes | No |
| Select a custom calibration function | Yes | Yes | Yes |
| Export processed data | Yes | Yes | Yes |
| **User Interface (UI)** | | | |
| UI for uploading data | Yes | Yes | No |
| UI for exporting data | Yes | Yes | No |
| UI for setting parameters | Yes | Yes | No |
| **UI for processed data** | | | |
| Table format | Yes | Yes | No |
| Thumbnails format | Yes | Yes | No |
| Thumbnails with details format | Yes | Yes | No |
| Sort data | Yes | Yes | No |
| Show the first derivative in thumbnails | Yes | No | No |
| Annotate faulty samples | Yes | No | No |
| Support to Copy table data | Yes | No | No |
| Support to Copy thumbnail image | Yes | No | No |
| **Detail Display of the samples growth curve** | | | |
| Display one sample | Yes | Yes | No |
| Display multiple (selection) samples | Yes | No | No |
| Zoom and Pan capabilities | Yes | No | No |
| Copy data | Yes | No | No |
| Copy Image | Yes | No | No |
