# Supplementary material for: PRECOG: a tool for automated extraction and visualization of fitness components in microbial growth phenomics
Source: BMC Bioinformatics. 2016 Jun 23;17:249. doi: 10.1186/s12859-016-1134-2 (PMC4917999; doi:10.1186/s12859-016-1134-2)
Supplement: Additional file 2: Figure S1. — Meta data to evaluate the extracted fitness components. Markers indicate data used for estimation of growth lag (purple circles), rate/doubling time (black cross), and efficiency (green triangles). Figures are screen shots from PRECOG. Figure S2. Displaying the first derivative of growth. X’s mark the samples where the doubling time was extracted, which coincides with the first derivative peak. Allows the user to identify curves where there are difficulties in extracting traits, like curves exhibiting multimodality (B and C). Figures are screenshots from PRECOG. Figure S3. PRECOG-lite website screenshots. (A) upload, B) table view, C) thumbnail view, D) a detail view of the growth-data, E) the Save As allows the user to save the data in its different forms (growth-data, first derivate, and extracted traits). Figure S4. High-quality benchmarking set of growth curves. The 100 growth curves of high quality displaying various growth feature, i.e. difference in growth lag, rate and efficiency as well as combinations of these. The set also include curves that are clearly multimodal. Red = raw data, Black = fully processed data. All curves are displayed on the log scale (y-axis). Figure S5. Low-quality benchmarking set of growth curves. The 100 growth curves of low quality, representing various technical challenges, e.g. curves with high levels of noise, frequent spikes and collapsing curves. Red = raw data, Black = fully processed data. All curves are displayed on the log scale (y-axis). Figure S6. First derivative of growth curves with various sampling frequencies. First derivative is displayed indicating times for measurements (red circles), and data used for estimation of growth rate (crosses). Figure S7. The effect of sampling frequency on growth lag (upper panel) and efficiency (lower panel). Sampling frequency denotes the fixed time (interval) between consecutive OD measurements. Averages from the high- and low-quality sets are indicated. (PDF 452 kb) [file 12859_2016_1134_MOESM2_ESM.pdf]

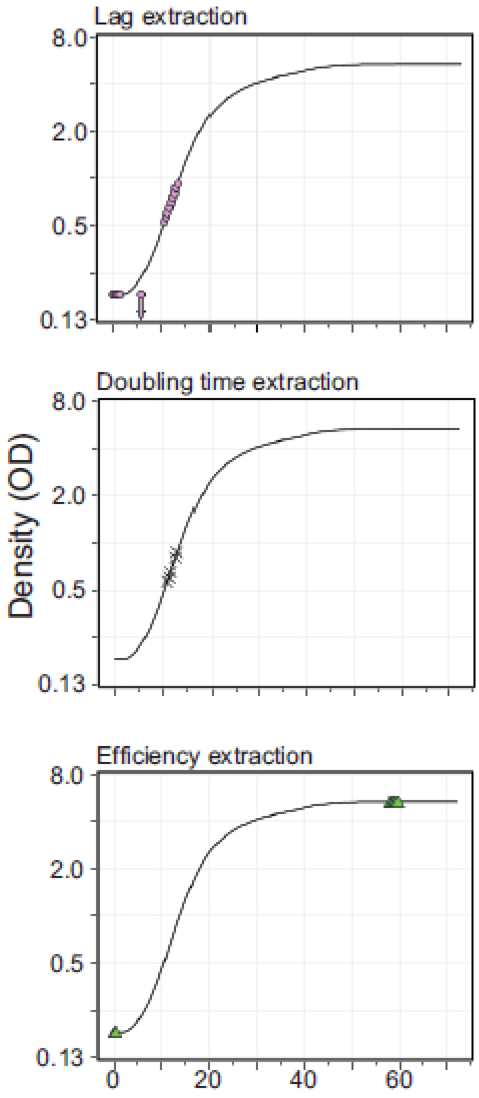

Fig S1 Fernandez-Ricaud *et al*

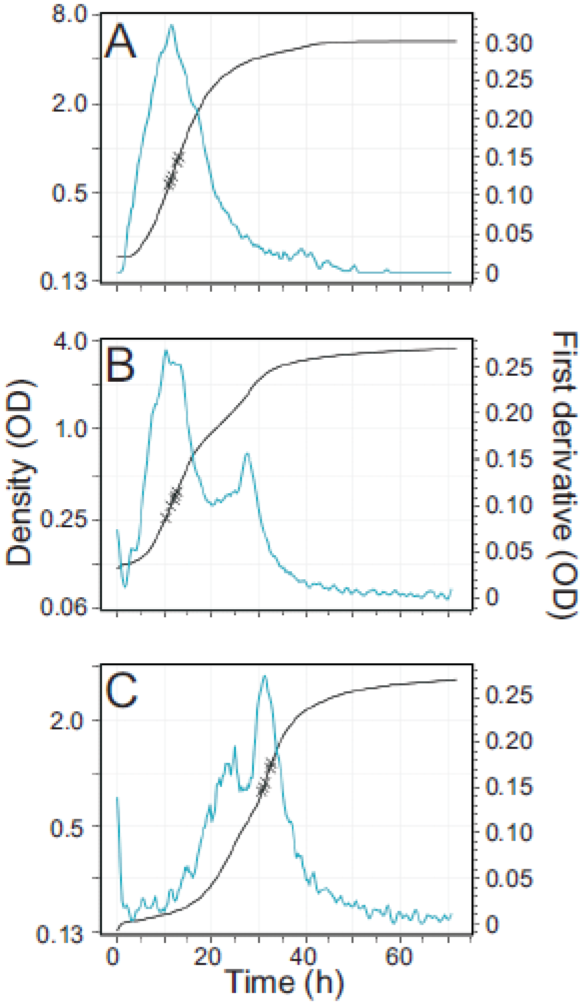

Fig S2 Fernandez-Ricaud *et al*

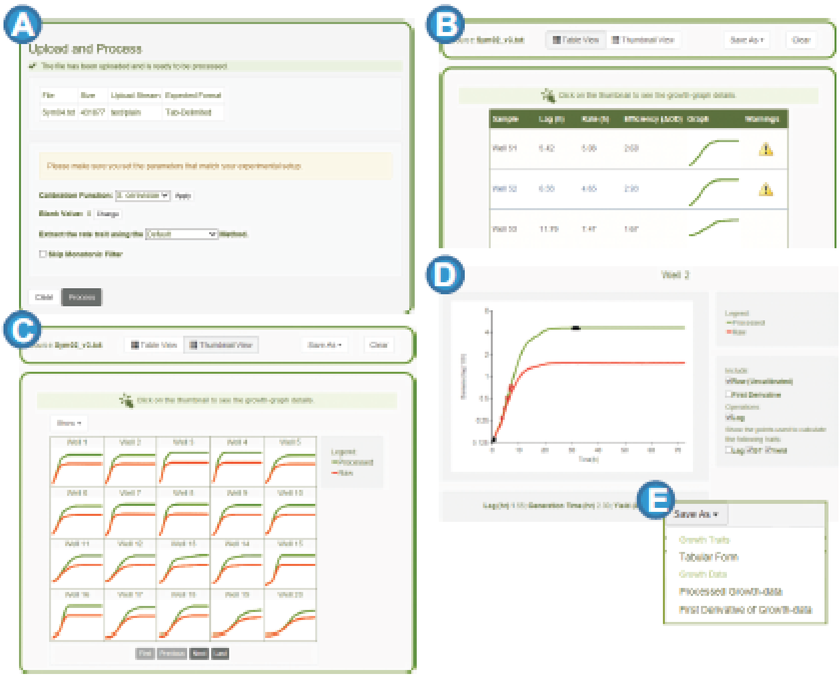

Fig S3 Fernandez-Ricaud *et al*

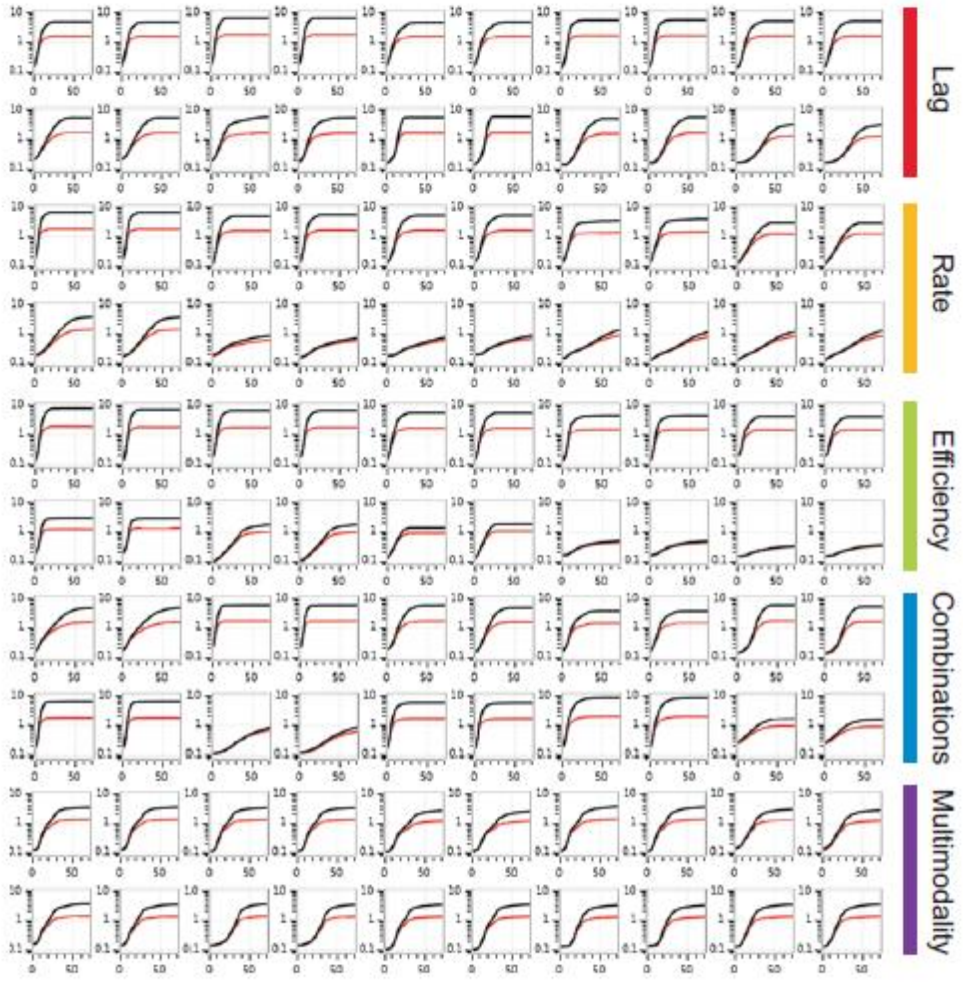

Fig S4 Fernandez-Ricaud *et al*

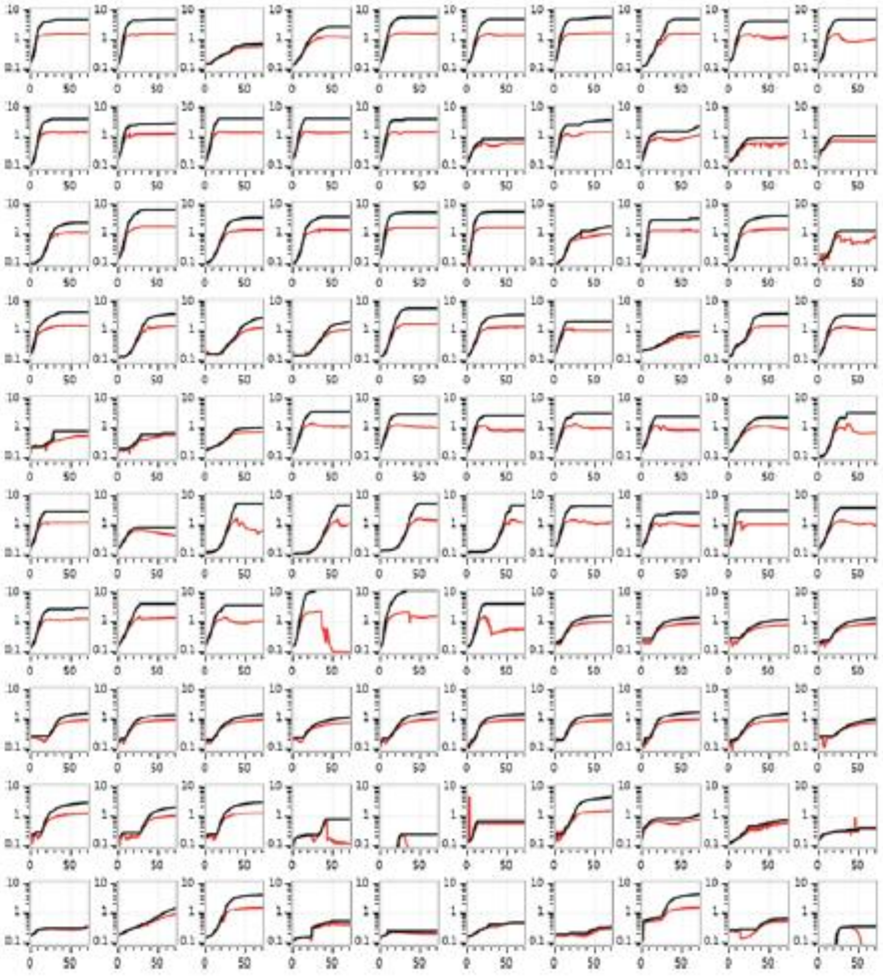

Fig S5 Fernandez-Ricaud *et al*

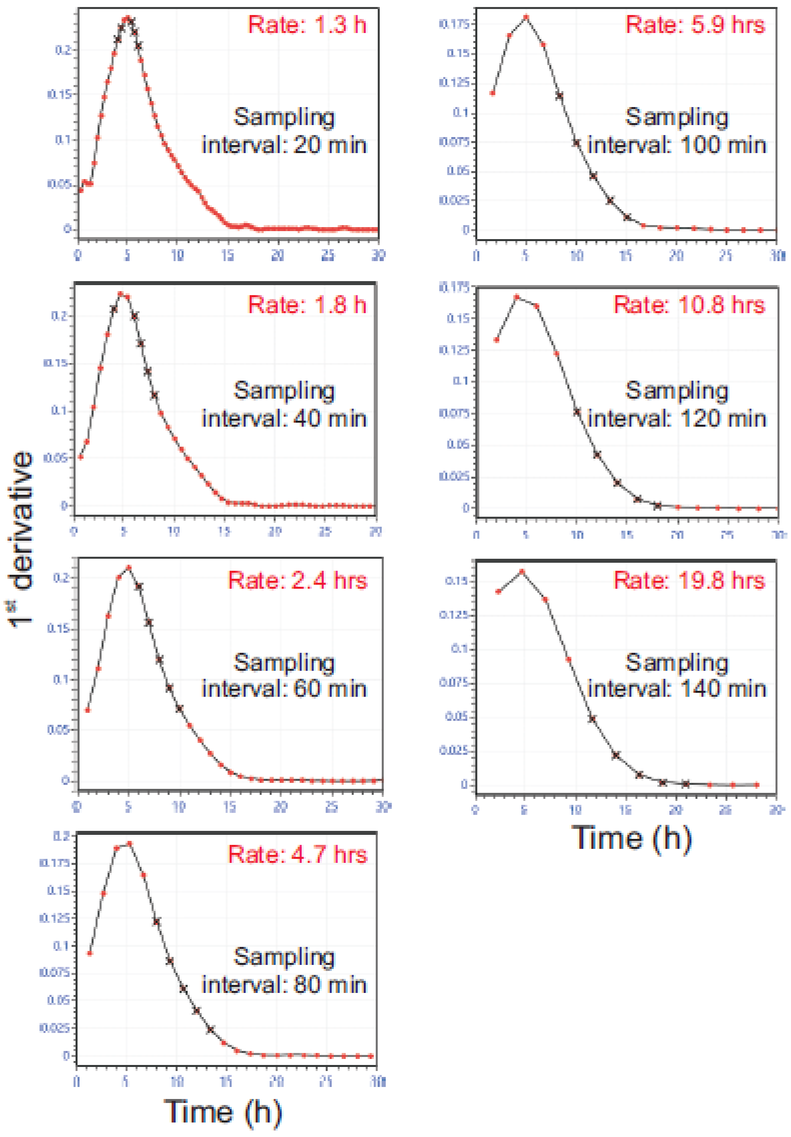

Fig S6 Fernandez-Ricaud *et al*

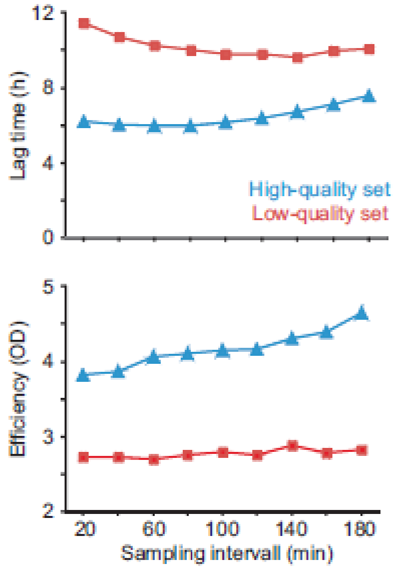

Fig S7 Fernandez-Ricaud *et al*
